# Mapping Viral Landscapes: Genomic Surveillance of Aedes Mosquitoes From Central India

**DOI:** 10.7759/cureus.79206

**Published:** 2025-02-18

**Authors:** Chitra Patankar, Umay Kulsum, Shruti Mahajan, Sudhir Kumar, Dipesh Kale, Vineet K Sharma, Shashwati Nema, Debasis Biswas

**Affiliations:** 1 Microbiology, All India Institute of Medical Sciences, Bhopal, Bhopal, IND; 2 Biological Sciences, Indian Institute of Science Education and Research, Bhopal, Bhopal, IND; 3 Biological Sciences, Indian Institute of Science Education and Research. Bhopal, Bhopal, IND

**Keywords:** mosquito metagenome, next generation sequencing, vector-borne disease, viral surveillance, virome analyses

## Abstract

Background: Mosquito-borne viral diseases pose a significant impact on human health, with the potential to cause widespread outbreaks of diseases. Monitoring viral genomes in mosquito populations can lead to informed risk assessment and promote early diagnosis. However, a standardized methodology is lacking to decipher circulating viral sequences in mosquito populations collected from human habitats. Our study aims to establish and evaluate a system of viral metagenomic analysis in the *Aedes *mosquito population.

Methods: Mosquitoes were collected using CDC-approved BG-Sentinel version 2 traps (Biogents AG, Regensburg, Germany) and battery-operated vacuum aspirators from different locations in the Bhopal region, India. They were sorted based on genus, gender, location, and collection date. The RNA was extracted from the homogenized mosquito pools and reverse transcribed. Complementary DNA (cDNA) was amplified using sequence-independent, single-prime amplification (SISPA). Further, polymerase chain reaction (PCR) products were sequenced using the Illumina NovaSeq 6000 platform (Illumina, Inc., San Diego, CA). Bioinformatic analysis of the reads was performed using Trimmomatic (Bolger AM, Lohse M, Usadel B (2014). Trimmomatic: A flexible trimmer for Illumina Sequence Data (Bioinformatics, btu170) for trimming low-quality raw reads. Later, Kraken2 and Bracken (Johns Hopkins University, Baltimore, MD) were used for the identification of viral sequences.

Results: The study examined virus diversity and seasonal distribution in mosquito populations collected from 13 sites in Bhopal, India, over 15 months. A total of 31 mosquito pools of female *Aedes *mosquitoes were analyzed. Metagenomic analysis revealed viruses encompassing plant, animal, insect, fungal, and bacteriophage hosts. The highest mosquito catch was during the post-monsoon period, while virus diversity peaked during the monsoon. Seasonal variations showed a higher frequency and diversity of viruses during the monsoon than during pre- and post-monsoon periods. The findings highlight the importance of temporal and ecological factors in viral surveillance and mosquito-borne disease management.

Conclusions: Our findings demonstrate the potential of combining entomological and genomic surveillance for monitoring virus circulation in mosquito populations, which may be implemented as a routine surveillance tool for the timely detection of spikes in viruses with human pathogenic potential and thus inform targeted vector control measures to avert potential outbreaks in the future.

## Introduction

Mosquito-borne diseases remain a significant global health burden, contributing significantly to morbidity and mortality, especially in tropical and subtropical regions [1]. Female *Aedes *mosquitoes play a pivotal role as vectors of arboviruses such as dengue, chikungunya, and Zika, which are responsible for widespread outbreaks worldwide [2]. Beyond the sheer burden of these diseases, the unpredictability of outbreaks poses a severe challenge, straining healthcare systems, particularly in resource-limited settings. In addition, the increasing incidence of diseases with zoonotic and transboundary potential [3,4] further underscores the need for continuous surveillance of vector populations.

Metagenomics has emerged as a promising tool for the comprehensive characterization of viral populations within a given sample source [5]. It allows for the identification of diverse viruses with minimal sample requirements, offering a powerful approach to understanding the virome of vector populations [6]. However, standardized protocols for metagenomic analyses of mosquito samples remain underdeveloped, limiting the consistent application of this method.

This study aims to address these challenges by exploring the potential of metagenomic sequencing of female *Aedes *mosquito populations collected across different seasons in Bhopal, India. By characterizing the virome of these populations, we aimed to gain critical insights into the circulating viral strains and their temporal dynamics. This approach could enhance our ability to predict disease outbreaks, develop targeted vector control strategies, and inform public health interventions. Our findings underscore the value of integrating genomic surveillance into routine monitoring systems, paving the way for proactive management of mosquito-borne diseases.

## Materials and methods

Sample collection

**Figure 1:**
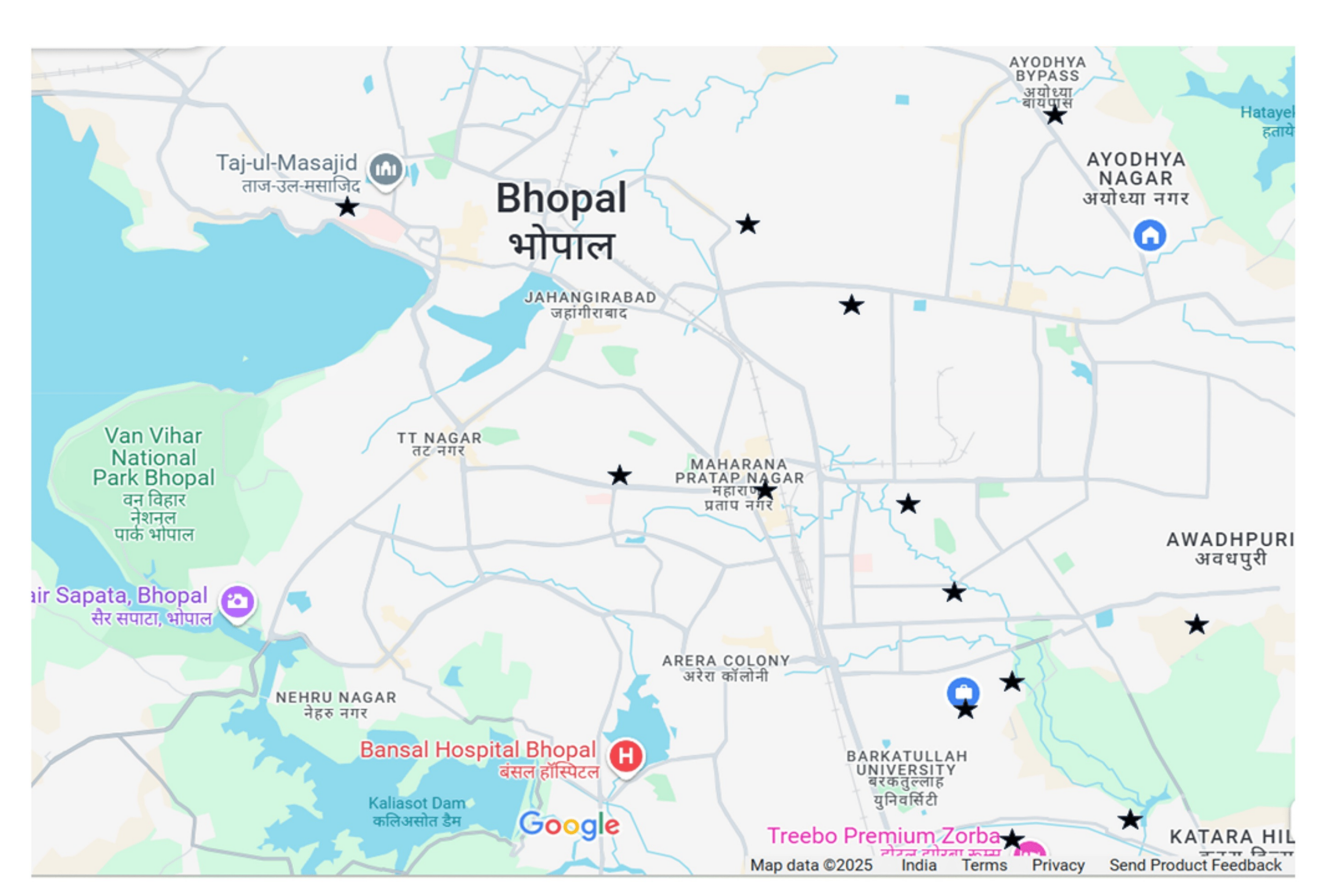
Locations selected for sample collection in the regional map of Bhopal district (n=13) Thirteen sites that were selected for the study are represented by black stars. Image credits: Map data ©2025 Google

Mosquito samples were collected from different locations in the Bhopal district (Figure 1) regularly at fortnightly intervals from July 2019 to September 2020. Sample collection was done using CDC-approved BG-Sentinel version 2 traps (Biogents AG, Regensburg, Germany) by placing the traps in indoor locations overnight. Battery-operated vacuum aspirators were used to collect the mosquitoes from the trap and immediately transferred to 4°C to freeze and anesthetize them. Mosquito gender and species identification was done using morphological characteristics and sorted on the basis of gender. They were stored in Qiagen RNAlater (Qiagen, Venlo, The Netherlands), overnight at 4°C, and then stored at -80°C till further processing.

Extraction of RNA and sequence-independent, single-prime amplification (SISPA)

The RNA was extracted from the mosquito pools by homogenizing them using Qiagen Tissue Lyser LT and Qiagen stainless steel beads (5 mm) (Qiagen) at 45 Hz for one minute. The mosquito debris were pelleted by spinning the homogenate at 10,000 rpm for 10 minutes. Further, 140 µl of supernatant was used for the RNA extraction using the RNesay mini kit (Qiagen) following the manufacturer’s protocol. Elution was done in 50 µl of nuclease-free water. Complementary DNA (cDNA) was synthesized using Superscript III reverse transcriptase (Thermo Fischer Scientific, Waltham, MA) and anchored random primers (Table 1) using the manufacturer’s protocol. To synthesize double-stranded cDNA, anchored random primers were added and incubated at 75°C for five minutes and then on ice for five minutes. Amplification was performed using the Klenow fragment at 37°C for 60 minutes, followed by 75°C for 10 min. To enhance the quantity of viral nucleic acid, SISPA was performed on the cDNA using GoTaq polymerase and barcode primers (Promega Corporation, Madison, WI) (Table 1) [7]. The amplification reaction was performed using the following conditions: 95°C for three minutes, 40 cycles of 95°C for 20 seconds, 54°C for 20 seconds, 68°C for 70 seconds, and a final extension at 68°C for seven minutes. Further polymerase chain reaction (PCR) products were purified using the QIAquick PCR purification kit (Qiagen).

**Table 1:** List of primers used for cDNA synthesis and SISPA cDNA: complementary DNA; SISPA: sequence-independent, single-prime amplification Source: [7]

	S.No.	Oligo name	Sequence
Anchored random primers	1	RT 1	GCCGGAGCTCTGCAGATATCNNNNNN
	2	RT 2	GTATCGCTGGACACTGGACCNNNNNN
	3	RT 3	ATCGTCGTCGTAGGCTGCTCNNNNNN
	4	RT 4	CGTAGATAAGCGGTCGGCTCNNNNNN
	5	RT 5	CATCACATAGGCGTCCGCTGNNNNNN
	6	RT 6	CGCAGGACCTCTGATACAGGNNNNNN
	7	RT 7	CGTCCAGGCACAATCCAGTCNNNNNN
	8	RT 8	CCGAGGTTCAAGCGAGGTTGNNNNNN
	9	RT 9	ACGGTGTGTTACCGACGTCCNNNNNN
	10	RT 10	CGACCCTCTTATCGTGACGGNNNNNN
	11	RT 11	GAGCCCCTAGACACAACGACNNNNNN
	12	RT 12	GGTGGGCGTGTGAAATCGACNNNNNN
	13	RT 13	GAAAATGAGAGGGGAGGCGGNNNNNN
Barcode primers	14	BP 1	GCCGGAGCTCTGCAGATATC
	15	BP 2	GTATCGCTGGACACTGGACC
	16	BP 3	ATCGTCGTCGTAGGCTGCTC
	17	BP 4	CGTAGATAAGCGGTCGGCTC
	18	BP 5	CATCACATAGGCGTCCGCTG
	19	BP 6	CGCAGGACCTCTGATACAGG
	20	BP 7	CGTCCAGGCACAATCCAGTC
	21	BP 8	CCGAGGTTCAAGCGAGGTTG
	22	BP 9	ACGGTGTGTTACCGACGTCC
	23	BP 10	CGACCCTCTTATCGTGACGG
	24	BP 11	GAGCCCCTAGACACAACGAC
	25	BP 12	GGTGGGCGTGTGAAATCGAC
	26	BP 13	GAAAATGAGAGGGGAGGCGG

Next-generation sequencing

The purified PCR products were taken for library preparation using the TruSeq DNA Nano library preparation kit (Illumina Inc., San Diego, CA). The final libraries were assessed on the Agilent TapeStation 4150 (Agilent Technologies, Inc., Santa Clara, CA) using high-sensitivity D1000 ScreenTapes (Agilent Technologies, Inc.) to check the library size. The libraries were quantified on the Qubit 4.0 fluorometer (Thermo Fischer Scientific) using the dsDNA high-sensitivity assay kit. The libraries were sequenced on the Illumina NovaSeq 6000 platform (Illumina Inc.) for 150 bp paired-end reads.

Data analysis

Preprocessing of raw sequence data was performed to remove low-quality sequences. Trimmomatic (0.39; Bolger AM, Lohse M, Usadel B. (2014). Trimmomatic: A flexible trimmer for Illumina Sequence Data (Bioinformatics, btu170) was used to remove TruSeq adapters and perform low-quality filtration by using a seed mismatch value of two and a maximum quality value of 30 for paired-end reads and 15 for single-end reads.

The translated search mode of Kraken2 (Johns Hopkins University, Baltimore, MD) was used to identify viral sequences in quality-filtered sequencing data. This method provides increased sensitivity in viral classification data. It was used to search reads from each sample to a database of Reference Sequence (RefSeq) viral proteins [8]. Later, Bracken (Johns Hopkins University) was used to calculate abundance at taxonomic levels. The relative abundance of viruses was calculated at family, genus, and species levels. Microbiome graphs were plotted using Pavian (FP Breitwieser, SL Salzberg - Bioinformatics, 2020).

## Results

A total of 13 representative sites were randomly selected for sample collection from within the city of Bhopal in central India (Figure 1). The sample collection spanned over 15 months, from July 2019 to September 2020. During this period, 34 mosquito pools containing female *Aedes* mosquitoes were collected. There was a mean (± SD) of eight (± 1) female *Aedes *mosquitoes per pool.

Temporal distribution of mosquito collection

We were interested in exploring the temporal distribution of the mosquito population in our region with reference to the seasonality of the monsoons. According to the definition adopted by the Indian Meteorological Department, we considered the months of February to May as the "pre-monsoon" period, June to September as the "monsoon" period, and October to January as the "post-monsoon" period [9].

**Figure 2:**
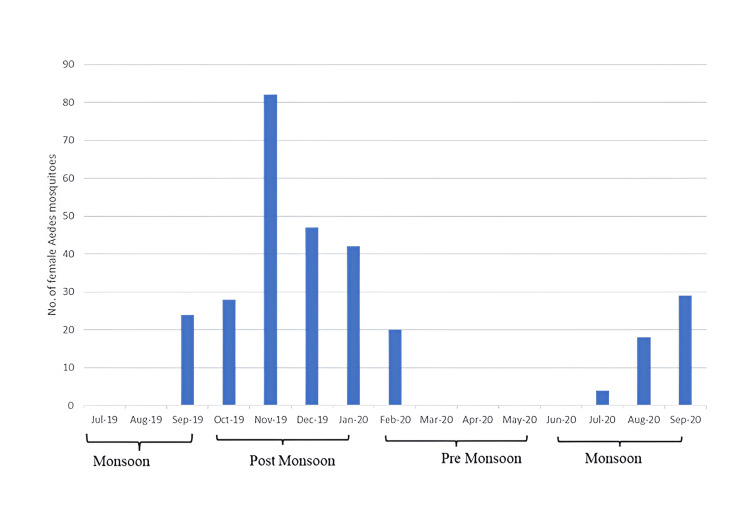
Temporal distribution of mosquito collection The x-axis represents months and years of mosquito sampling. The format "MMM-YY" indicates the month (e.g., "Jul" for July) and the last two digits of the year (e.g., "19" for 2019; "20" for 2020). The y-axis shows the number of *Aedes* mosquitoes collected during each period. This image has been created by the author using Microsoft Excel (Microsoft Corp., Armonk, NY).

The maximum catchment of *Aedes *mosquitoes was noted in the post-monsoon period, compared to the pre-monsoon and monsoon periods (Figure 2).

Read quality

**Figure 3:**
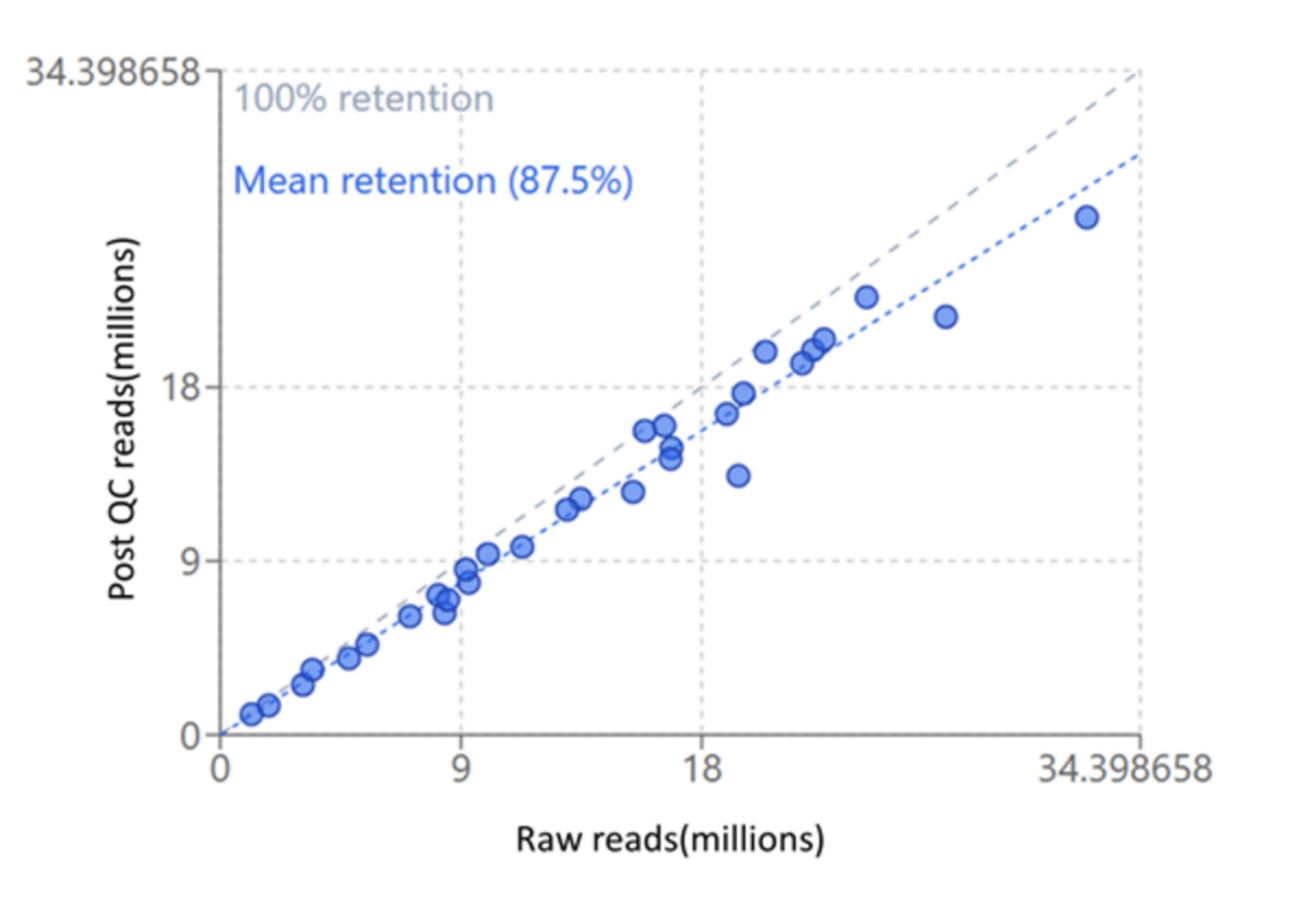
Sequencing quality control (QC) efficiency across samples X-axis: raw reads (millions); y-axis: post-QC reads (millions) This image has been created by the author using Microsoft Excel (Microsoft Corp., Armonk, NY).

The quality of the reads was evaluated using FastQC (The Babraham Institute, Cambridge, UK), and only those with an average Phred score exceeding 36 were retained for further analysis. Reads with an average Phred score below 36 were excluded from subsequent steps. Before quality control (QC), the raw sequencing depth varied considerably among samples, ranging from approximately 1.2 million to 32.4 million paired-end reads. After applying QC filters, read counts were reduced to between 1.1 million and 26.8 million reads, with a mean retention rate of 87.5% (Figure 3).

Metagenome sequencing

In this study, 428,276,613 paired-end reads for all pools were generated, with a mean (± SD) of 13,815,374.61 (± 7,917,625.22) reads per pool. After quality filtration, 375,010,668 reads remained, with a mean (± SD) of 12,097,118.32 (± 6,927,746.71) quality-filtered reads per pool. These were classified in Kraken2 to identify viral reads, wherein the mean percentage of classified viral reads was 0.218% with a standard deviation of 0.157% across all the pools. The mean percentage of unclassified reads was 99.78%, with a standard deviation of 0.157%.

**Figure 4:**
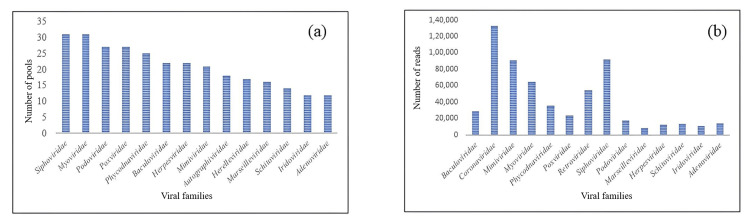
(a) Distribution of different families across pools; (b) Abundance of reads across different viral families This figure has been created by the authors using Microsoft Excel (Microsoft Corp., Armonk, NY).

Analyzing the distribution of the viral families in the classified viral reads, we observed the absence of a perfect overlap between the pool-wise prevalence of viral families and the abundance of their reads. While the *Siphoviridae *family was the most prevalent, being detected in all the pools, the *Coronaviridae *family contributed to the maximum number of viral reads (132,789). The distribution of the different families across different pools and the abundance of their viral reads are shown in Figure 4a and Figure 4b.

Distribution of viral reads obtained from sequencing

The taxonomic evaluation of the reads from different viral families led to the identification of 310 genera and 423 species. Interestingly, in agreement with the feeding habits of the *Aedes *mosquitoes, we found viral sequences of diverse host populations in the mosquito samples, including plant, animal, insect, fungal, and bacteriophage viruses.

**Figure 5:**
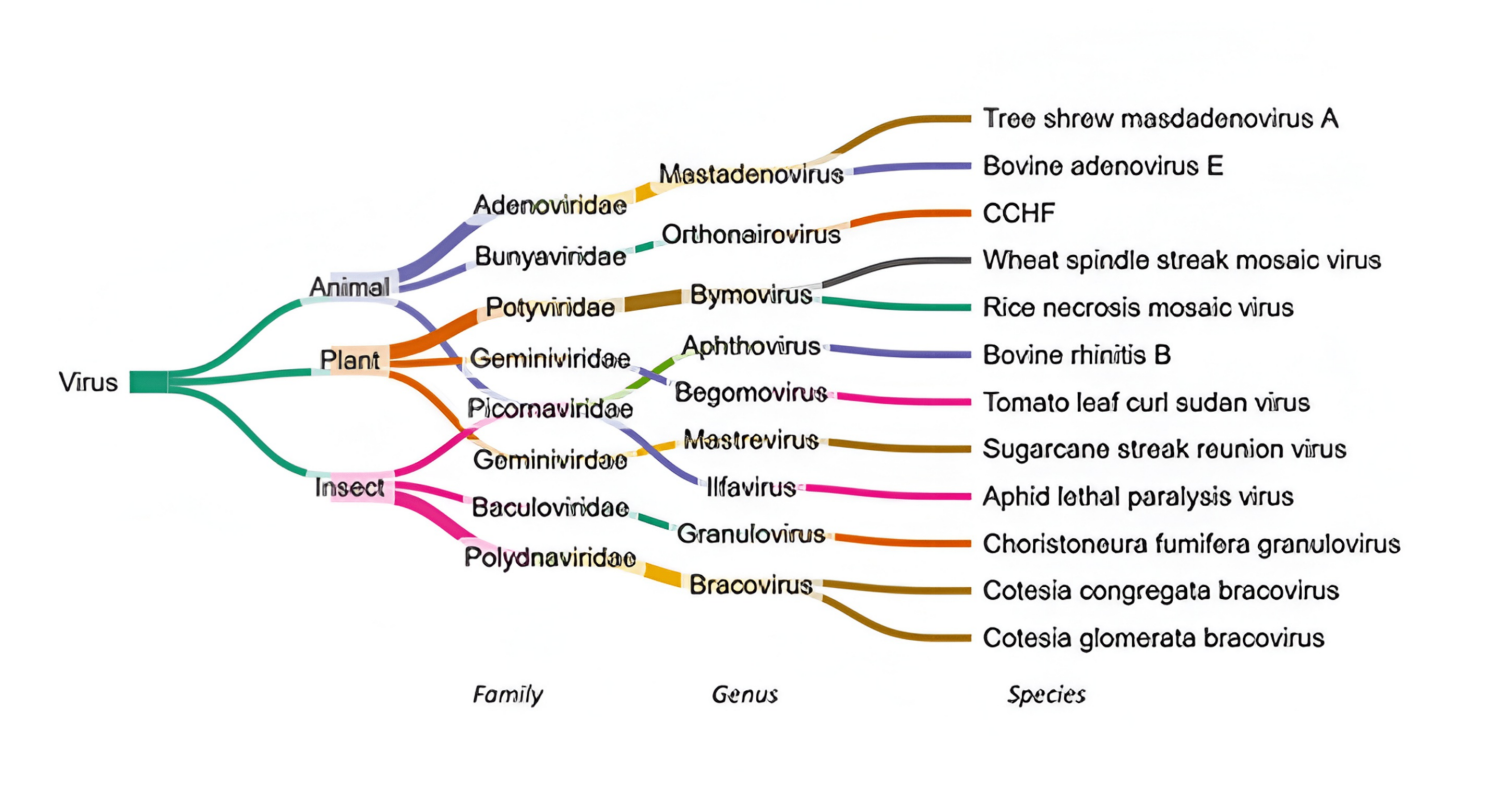
Taxonomic distribution of different viruses identified in this study Hierarchical classification of viruses based on their host categories: animal, plant, and insect. Each branch corresponds to a taxonomic level: family, genus, and species, progressing from left to right. The first division groups viruses based on their primary hosts: animal (purple), plant (orange), and insect (pink). Each host category splits further into viral families, genera, and species. The branches are colored uniquely to aid distinction. CCHF: Crimean-Congo hemorrhagic fever This figure has been created by the authors using the SankeyMATIC (produced by Steve Bogart (@nowthis@tilde.zone)).

Among the plant viruses, several notable pathogens from the *Bymovirus *genus in the *Potyviridae *family were detected, including Wheat spindle streak mosaic virus, which affects wheat grain yields, and Rice necrosis mosaic virus, which infects rice plants. Additionally, the Tomato leaf curl Sudan virus (genus *Begomovirus*, family *Geminiviridae*), previously observed in various agroclimatic zones of Gujarat, India [10], was identified in our mosquito pools. The presence of sugarcane streak reunion virus (genus *Mastrevirus*, family *Geminiviridae*) was also recorded, further expanding the spectrum of plant viruses detected. Other plant-associated viruses, including Mirabilis jalapa mottle virus and Kyuri green mottle mosaic virus, were also identified in the mosquito pools (Figure 5).

Among the animal viruses identified in our mosquito pools, we observed Tree shrew mastadenovirus A in seven pools. Tree shrew mastadenovirus A is a type of virus that can infect tree shrews, leading to respiratory diseases. Mastadenoviruses belong to the family *Adenoviridae*, and they are associated with illnesses in animals and humans, commonly affecting respiratory and gastrointestinal systems. Crimean-Congo hemorrhagic fever (CCHF) virus was observed in only one pool, a tick-borne virus of the family *Bunyaviridae*. The other viruses with the animal pathogenic potential present in only a single pool are Bovine adenovirus E and Bovine rhinitis B. Murine leukemia virus of the genus *Gammaretrovirus* was observed in nine pools (Figure 5).

Choristoneura fumiferana granulovirus, an insect virus of the genus *Granulovirus *and family *Baculoviridae*, was observed in seven pools. This virus infects the spruce budworm (*Choristoneura fumiferana*) and is a potential agent for pest management in forestry. Aphid lethal paralysis virus of the genus Iflavirus and family *Picornaviridae* was detected in one pool; this virus is pathogenic to aphids and causes paralysis, impacting aphid populations that are key agricultural pests [11]. We also found reads of non-pathogenic insect viruses such as Cotesia congregate bracovirus and Cotesia glomerata bracovirus of the genus *Bracovirus* and family *Polydnaviridae *in two mosquito pools. These non-pathogenic viruses infect parasitoid wasps and are used in biological control. Wuhan mosquito virus 9, detected in a single pool, is a lesser-known virus from mosquito populations, and its pathogenic potential remains to be fully understood. Cell fusing agent virus (CFAV) of the family *Flaviviridae*, a promising biocontrol agent for preventing mosquito-borne diseases as it negatively impacts the growth of arboviruses [12], was detected (Figure 5).

Giant viruses were also found in our mosquito pools. Acanthamoeba polyphaga mimivirus of the genus *Mimivirus *was the most abundant virus found in 15 out of 31 mosquito pools. Cafeteria roenbergensis virus was found in eight mosquito pools, which was followed by *Pandoravirus neocaledonia* detected in five pools.

In addition, algal viruses identified in our mosquito pools included Paramecium bursaria Chlorella virus in five mosquito pools, Chrysochromulina ericina virus in four mosquito pools, and Dishui lake phycodnavirus 1 in two mosquito pools. Fungal viruses *Feldmannia *species virus in two pools, *Sclerotinia sclerotiorum* dsRNA mycovirus-L, Britarnavirus 1, and Ceratobasidium endornavirus B in single pools.

Seasonal distribution of viruses

**Figure 6:**
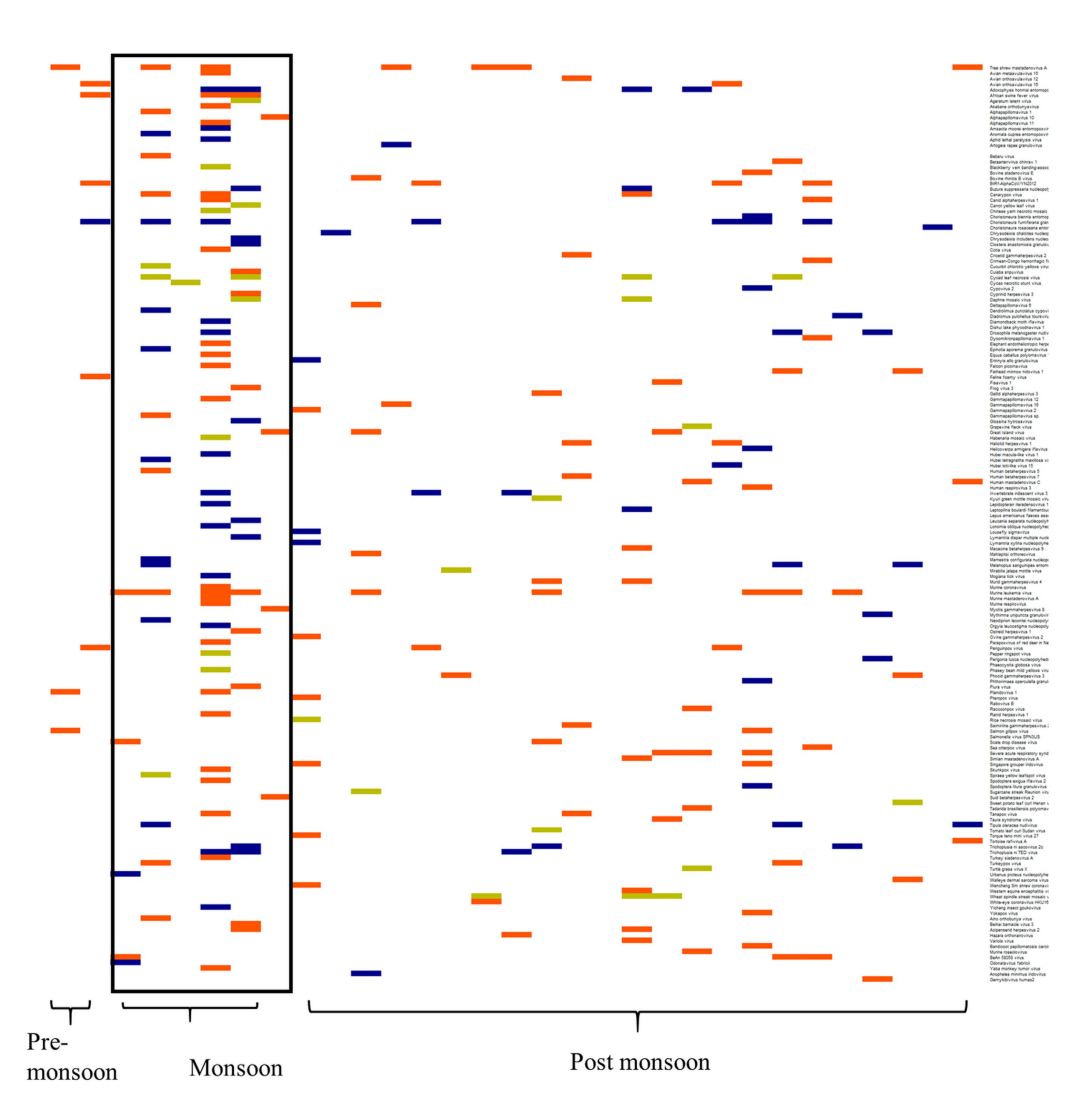
Seasonal variations in virus distribution across different hosts The X-axis represents the mosquito pools. The Y-axis represents identified viral species of different host categories (plant: orange, animal: green, insect: blue) during three distinct seasonal phases: pre-monsoon, monsoon, and post-monsoon The heatmap has been created by the authors using the R package (Galili, Tal, O'Callaghan, Alan, Sidi, Jonathan, Sievert, Carson (2017). “heatmaply: an R package for creating interactive cluster heatmaps for online publishing.” Bioinformatics. doi:10.1093/bioinformatics/btx657, https://academic.oup.com/bioinformatics/article-pdf/doi/10.1093/bioinformatics/btx657/21358327/btx657.pdf.)

We analyzed the seasonal distribution of viruses with pathogenic potential in plant, animal, and insect hosts and observed wide seasonal variation in their circulation. Though a higher number of mosquito pools was collected in the post-monsoon period, wider diversity and higher frequency of all three types of viruses were observed in the monsoon period compared to the other two periods (p<0.005) (Figure 6).

## Discussion

Our comprehensive metagenomic analysis of female *Aedes *mosquito populations in Bhopal, India, revealed a remarkably diverse viral ecosystem comprising 73 families distributed across 310 genera and 423 species. This extensive viral diversity spans multiple host categories, including vertebrates, invertebrates, plants, and microorganisms, highlighting the complex ecological role of *Aedes* mosquitoes as viral repositories. The predominant viral families identified in our study were *Siphoviridae *and *Myoviridae*, detected consistently across all 31 mosquito pools. Notably, the *Coronaviridae *family emerged as the most abundant in terms of read count (132,789 reads), followed by *Siphoviridae *(91,815 reads) and *Mimiviridae *(90,999 reads).

Our findings align with previous studies reporting diverse mosquito-associated viromes, such as those by Ng et al. and Nebbak et al., who identified bacteriophages and giant viruses, respectively, in mosquito populations [13,14]. Interestingly, we observed a higher prevalence of animal and insect viruses compared to plant viruses, consistent with reports from Hameed et al. and Gangopadhyay et al. Geographic and ecological variations likely contributed to differences in viral species identified across studies [15,16]. Hameed et al. also observed less circulation of plant viruses than insect and animal viruses in *Aedes *mosquito populations [15]. In another study by the same author, animal viruses were reported in different viral families [17]. Khan et al. also noticed that mosquito populations increase after rainfall [18]. 

Our temporal analysis and seasonal analysis revealed a striking pattern: despite higher mosquito abundance during the post-monsoon period, viral diversity and circulation peaked during the monsoon season. This pattern was statistically significant across all host categories (p<0.005), suggesting that environmental factors during the monsoon season may create optimal conditions for viral transmission and persistence within mosquito populations. These findings too are in agreement with previous studies reporting similar seasonal patterns in mosquito abundance and viral prevalence [19].

Notably, we observed no viruses of direct human pathogenic potential, which correlates with the relatively low incidence of mosquito-borne diseases in the region during our study period (2019-2020). This observation is consistent with findings from other recent studies by Liu et al. and Kubacki et al., who also reported an absence of known pathogenic arboviruses in their mosquito surveillance [20,21].

The identification of a diverse virome within *Aedes *mosquitoes has significant implications for public health and pest management. The presence of insect and plant viruses in mosquito populations suggests potential applications in biological control strategies, such as using specific viruses to selectively target pest species. Furthermore, the detection of pathogenic animal viruses underscores the need for mosquito-based surveillance systems to predict and mitigate potential zoonotic spillovers. These insights highlight the feasibility of integrating virome analysis into routine vector surveillance programs, particularly during periods of heightened viral circulation, such as the monsoon season.

Our study suffered from several limitations, primarily the low abundance and retrieval rates of viral reads, which impacted the assembly and annotation of certain sequences. The inability to detect certain viruses in more than one pool limited our capacity to confirm their regional circulation. Additionally, the lack of a standardized protocol for mosquito-derived viral nucleic acids and interference from environmental factors, such as insecticide spraying, posed challenges. Future studies should explore methods to improve viral read recovery, such as optimized sampling techniques and advanced sequencing platforms. Expanding the geographical and temporal scope of sampling, along with characterizing unclassified viral reads, could reveal novel viruses and enhance our understanding of mosquito viromes.

## Conclusions

Our findings demonstrate the potential of combining entomological and genomic surveillance for monitoring virus circulation in mosquito populations. Such systems could be instrumental in monitoring virome dynamics, predicting potential zoonotic spillovers, and guiding targeted pest and disease control strategies, and thus prove useful for early detection of emerging pathogens and potential disease outbreaks. The seasonal patterns we observed in viral diversity and abundance could inform the timing and intensity of vector control measures. Further investigations with expanded geographic and temporal sampling, coupled with optimized sequencing methodologies, are essential for advancing our understanding of mosquito-associated viromes and their implications for public health.
